# Descenso da Pressão Arterial durante o Sono e o Sistema Nervoso Autônomo

**DOI:** 10.36660/abc.20200280

**Published:** 2020-05-12

**Authors:** Fernando Antonio de Almeida

**Affiliations:** Departamento de Clínica Faculdade de Ciências Médicas e da Saúde PUC-SP Sorocaba SP Brasil Departamento de Clínica da Faculdade de Ciências Médicas e da Saúde da PUC-SP, campus Sorocaba, Sorocaba, SP – Brasil

**Keywords:** Doença de Chagas, Sistema Nervoso Autônomo, Monitoração Ambulatorial da Pressão Arterial (MAPA, Pressão Arterial, Sono, Diabetes Mellitus

A pressão arterial (PA) é controlada continuamente por mecanismos complexos que envolvem as características estruturais do sistema arterial, o sistema nervoso autônomo (simpático e parassimpático) integrado aos sistemas de barorreceptores e quimiorreceptores, o volume circulante e vários sistemas hormonais vasoconstritores e vasodilatadores com ações sistêmicas e locais.^1^ A integração desses sistemas garante que a pressão arterial sofra mínimas variações em intervalos pequenos; no entanto, se considerarmos todo o dia, há momentos (p. ex., durante o sono e ao levantar-se pela manhã) em que ocorrem variações mais intensas da PA, sempre em torno de uma média. A monitoração ambulatorial da pressão arterial (MAPA) permite registrar esse fenômeno na prática clínica.

**Figura 1:**
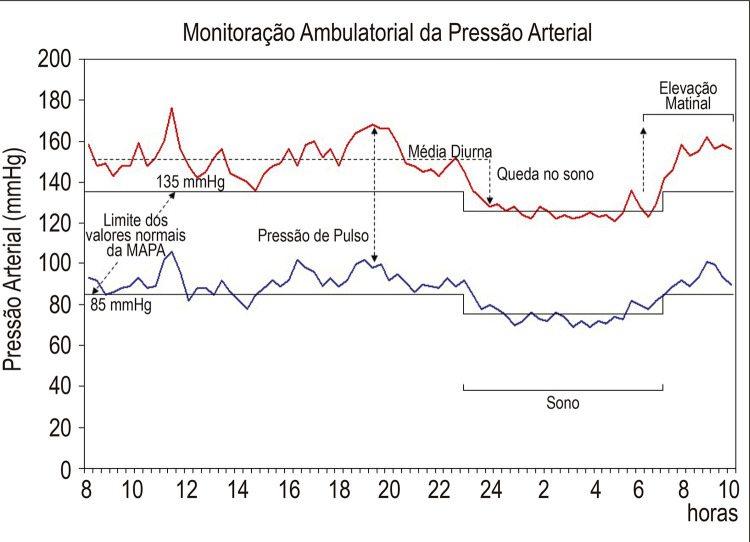
– *Registro gráfico da monitorização ambulatorial da pressão arterial de uma pessoa com hipertensão arterial. Observe a queda pressórica durante o sono e a elevação matinal da pressão arterial.*

A Figura 1 apresenta o registro gráfico da MAPA de uma pessoa com hipertensão arterial indicando os principais parâmetros avaliados nesse exame. Um dos fenômenos mais importantes que pode ser avaliado por MAPA é a redução fisiológica da PA durante o sono. Esse comportamento fisiológico da PA durante o sono ocorre porque muitos mecanismos vasoconstritores são “desarmados” nessa condição – entre eles, o sistema nervoso autônomo é um dos mais importantes.^2^ Uma consequência direta desse efeito modulador do sistema nervoso autônomo é que, nas doenças ou condições clínicas em que está comprometido, a ausência desse efeito modulador se expressa pela ausência do descenso da PA durante o sono. Em alguns casos, pode haver, inclusive, elevação da PA durante o sono. Este é o exemplo clássico de indivíduos com *diabetes mellitus* com neuropatia autonômica:^3 - 5^ eles frequentemente apresentam hipotensão postural, elevação da PA ao deitar-se e ausência do descenso da PA durante o sono.^3 , 4 , 6 , 7^ A ausência do descenso da PA durante o sono implica maior carga pressórica sobre o sistema circulatório e aumenta o risco de eventos cardiovasculares a longo prazo.^8 , 9^ Existem outras condições clínicas associadas à ausência do descenso da PA durante o sono, mas não é o caso desta discussão.

No artigo original publicado neste número dos *Arquivos Brasileiros de Cardiologia,* ^10^ com base na estratégia metodológica o estudo caso-controle, os autores constataram, por meio de MAPA, que 54 adultos (30 mulheres, com média de idade de 36 anos) com doença de Chagas aguda transmitida por via oral têm maior prevalência da ausência do descenso da PA durante o sono (74%) e maior prevalência de ascensão da PA durante o sono (18,5%). A frequência com que essas alterações aparecem nos participantes do estudo com doença de Chagas aguda foi significantemente superior em comparação aos participantes do grupo-controle, sendo, respectivamente, 16,6% e 1,8%. A elevação da PA durante o sono é também uma característica dos pacientes com *diabetes mellitus* .^7^

Os autores identificaram que essas alterações são precoces na doença de Chagas aguda e interpretaram que a tais alterações na MAPA possam decorrer da desautonomia, característica da doença de Chagas crônica, e que já está presente na fase aguda da doença. O estudo é uma importante contribuição para o conhecimento na área, visto que produz uma documentação contundente de alterações funcionais do sistema nervoso autônomo nas fases iniciais da doença de Chagas.^10^ Uma questão que imediatamente se impõe é saber se o tratamento da doença de Chagas na fase aguda pode impedir a progressão ou recuperar as lesões neurológicas já estabelecidas. A última diretriz brasileira sobre doença de Chagas menciona como critérios de cura a ausência de parasitemia e a redução dos títulos de anticorpos ao longo de 5 a 10 anos, mas não aborda esse aspecto da doença.^11^ Os autores do presente estudo têm a oportunidade de acompanhar esses pacientes por períodos prolongados, a fim de avaliar se o tratamento da doença de Chagas aguda poderá modificar a evolução das lesões do sistema nervoso autônomo.
