# Supplementary material for: Descenso da Pressão Arterial durante o Sono e o Sistema Nervoso Autônomo
Source: Arq Bras Cardiol. 2020 May 12;114(4):716–7. [Article in Portuguese] doi: 10.36660/abc.20200280 (PMC9744351; doi:10.36660/abc.20200280)
Supplement: Translation

# Translation: Nocturnal Blood Pressure Dipping and the Autonomic Nervous System

**Authors:** Fernando Antonio de Almeida

**Affiliations:** Brazil Departamento de Clínica da Faculdade de Ciências Médicas e da Saúde da PUC-SP, campus Sorocaba, Sorocaba, SP – Brazil

**Keywords:** Chagas Disease, Autonomic Nervous System, Blood Pressure Monitoring Ambulatory, Blood Pressure, Sleep, Diabetes Mellitus

Blood Pressure (BP) is continuously controlled by complex mechanisms involving the structural characteristics of the arterial system, the autonomic nervous system (sympathetic and parasympathetic) integrated with the baroreceptor and chemoreceptor systems, the circulating volume and several vasoconstrictor and vasodilator hormone systems with systemic and local actions.^1^ The integration of these systems ensures that the blood pressure undergoes minimal variations in small intervals, but if we consider the whole day, there are times, such as during sleep and when getting up in the morning, when there are more intense variations in blood pressure, always around mean values. Ambulatory blood pressure monitoring (ABPM) allows this phenomenon to be recorded in clinical practice.

Figure 1 shows the ABPM graphical record of a person with arterial hypertension, indicating the main parameters evaluated in this exam. One of the most important phenomena that can be assessed by ABPM is the physiological BP dipping during sleep. This physiological behavior of BP during sleep occurs because many vasoconstrictor mechanisms are “disarmed” in this condition; among them, the autonomic nervous system is one of the most important.^2^ A direct consequence of this modulatory effect of the autonomic nervous system is that, in diseases or clinical conditions in which the system is affected, the absence of this modulatory effect is expressed by the absence of BP dipping during sleep. In some cases, there may even be an increase in BP during sleep. This is the classic example of individuals with diabetes mellitus with autonomic neuropathy.^3 - 5^ These individuals frequently have postural hypotension, elevated BP at bedtime and absence of BP dipping during sleep.^3 , 4 , 6 , 7^ The absence of BP dipping during sleep implies a higher pressure load on the circulatory system and increases the risk of cardiovascular events in the long-term.^8 , 9^ There are other clinical conditions associated with the absence of BP dipping during sleep, but this is not the case of this discussion.

**Figure 1:** – *Graphical record of an ambulatorial blood pressure monitoring of a person with hypertension. Observe nocturnal dipping and morning surge.*

In the original article published in this issue of *Arquivos Brasileiros de Cardiologia,* ^10^ using the case-control study as a methodological strategy, the authors documented by ABPM that 54 adults (30 women, mean age 36 years) with acute Chagas disease transmitted orally have a higher prevalence of the absence of BP dipping during sleep (74%) and higher prevalence of BP increase during sleep (18.5%). The frequency with which these changes occur in the study participants with acute Chagas’ disease was significantly higher when compared to participants in the control group, respectively 16.6% and 1.8%. The increase in BP during sleep is also a characteristic of patients with diabetes mellitus.^7^

The authors identified that these alterations occur early in acute Chagas’ disease and interpreted that such alterations in ABPM may result from dysautonomia, a characteristic of chronic Chagas’ disease, which is already present in the acute phase of the disease. The study is an important contribution to the knowledge in the area, as it produces a remarkable documentation of functional alterations in the autonomic nervous system in the early stages of Chagas’ disease.^10^ A question that immediately arises is whether the treatment of Chagas’ disease in the acute phase can prevent the progression of or recover the already established neurological lesions. The latest Brazilian guideline on Chagas’ disease mentions the absence of parasitemia and the reduction in antibody titers over 5 to 10 years as the cure criteria but does not address this aspect of the disease.^11^ The authors of the present study have the opportunity to monitor these patients for prolonged periods of time to assess whether the treatment of acute Chagas’ disease can modify the evolution of the autonomic nervous system lesions.
